# Supplementary material for: Mitogen and Stress-Activated Kinases 1 and 2 Mediate Endothelial Dysfunction
Source: Int J Mol Sci. 2021 Aug 11;22(16):8655. doi: 10.3390/ijms22168655 (PMC8395442; doi:10.3390/ijms22168655)
Supplement: Supplementary file 1 [file ijms-22-08655-s001.zip › ijms-1277274-supplementary.pdf]

Supplemental Materials:

Supplement 1: Activation of dermal microvascular endothelial cells and detection of MSK1, CREB, P38, ERK, HSP27 and MK2 by western blot analysis.

Supplement 2: Activation of dermal microvascular endothelial cells using recombinant mouse TNF-α and detection of MSK1, CREB, P38, ERK, HSP27 and MK2 by western blot analysis.

Supplement 3: Activation of dermal microvascular endothelial cells using anisomycin and detection of MSK1, CREB, P38, ERK and MK2 by western blot analysis.

Supplement 4: Body weight of MSK1/2 knock-out and wild-type (WT) mice at study (A) baseline (A) and (B) after 24 weeks on standard chow or a cholesterol enriched diet. A and B were analysed by an unpaired T-test.

Supplement 5: Body weight of MyD88 knock-out and wild-type (WT) mice at study (A) baseline and (B) after 24 weeks on standard chow or a cholesterol enriched diet. A and B were analysed by an unpaired T-test.

Supplementary Data S1

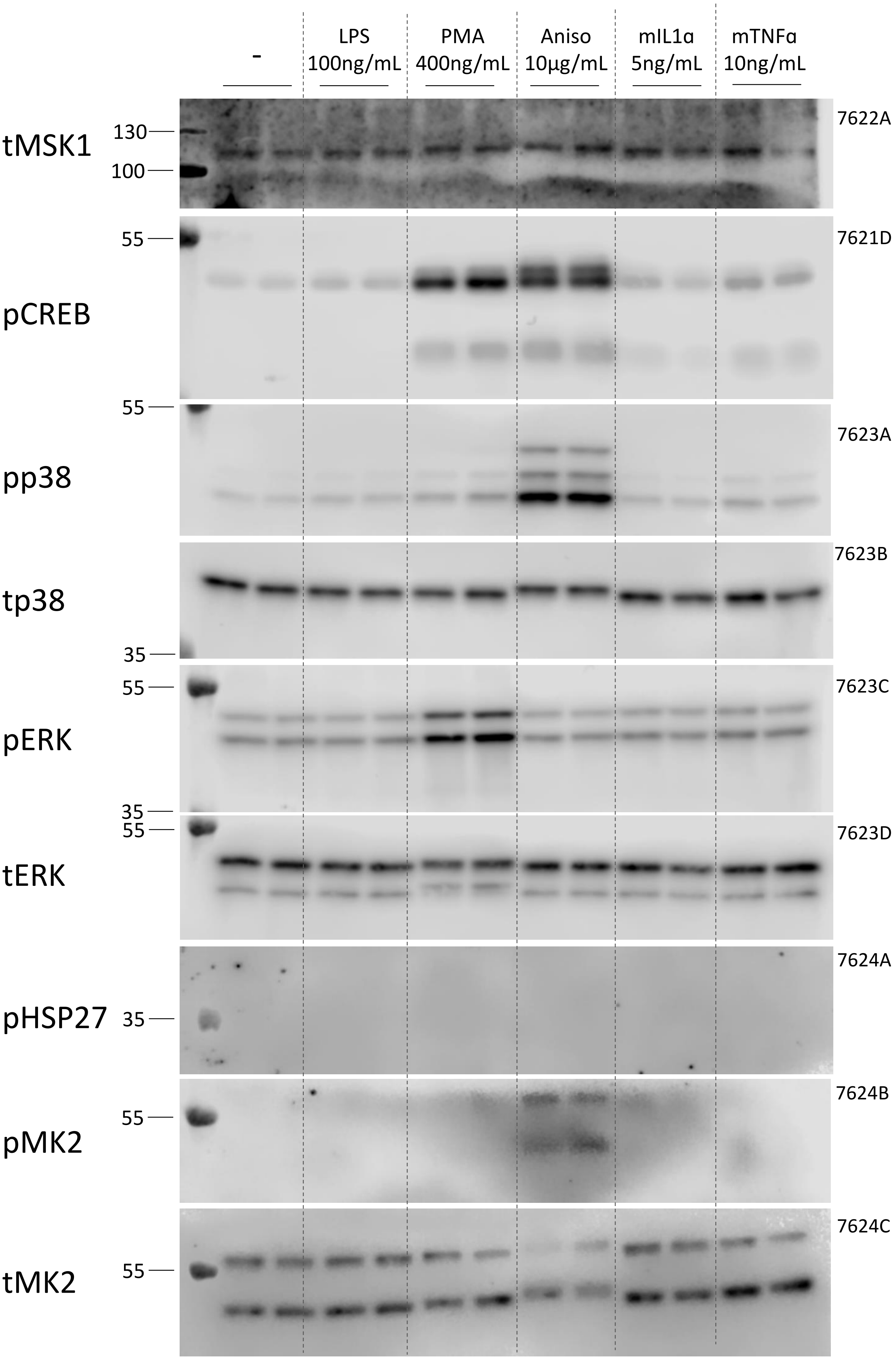

Supplementary Data S2

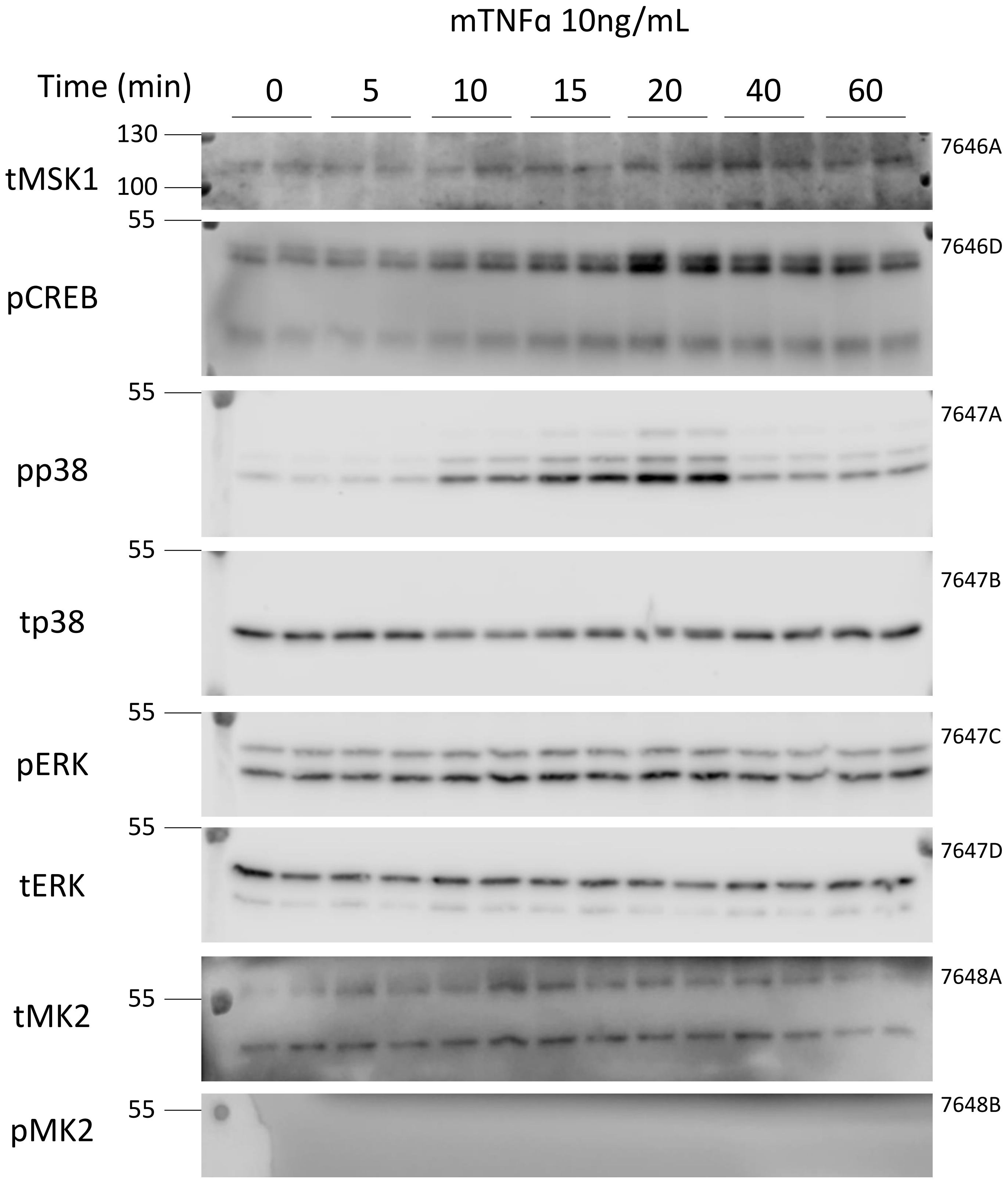

Supplementary Data S3

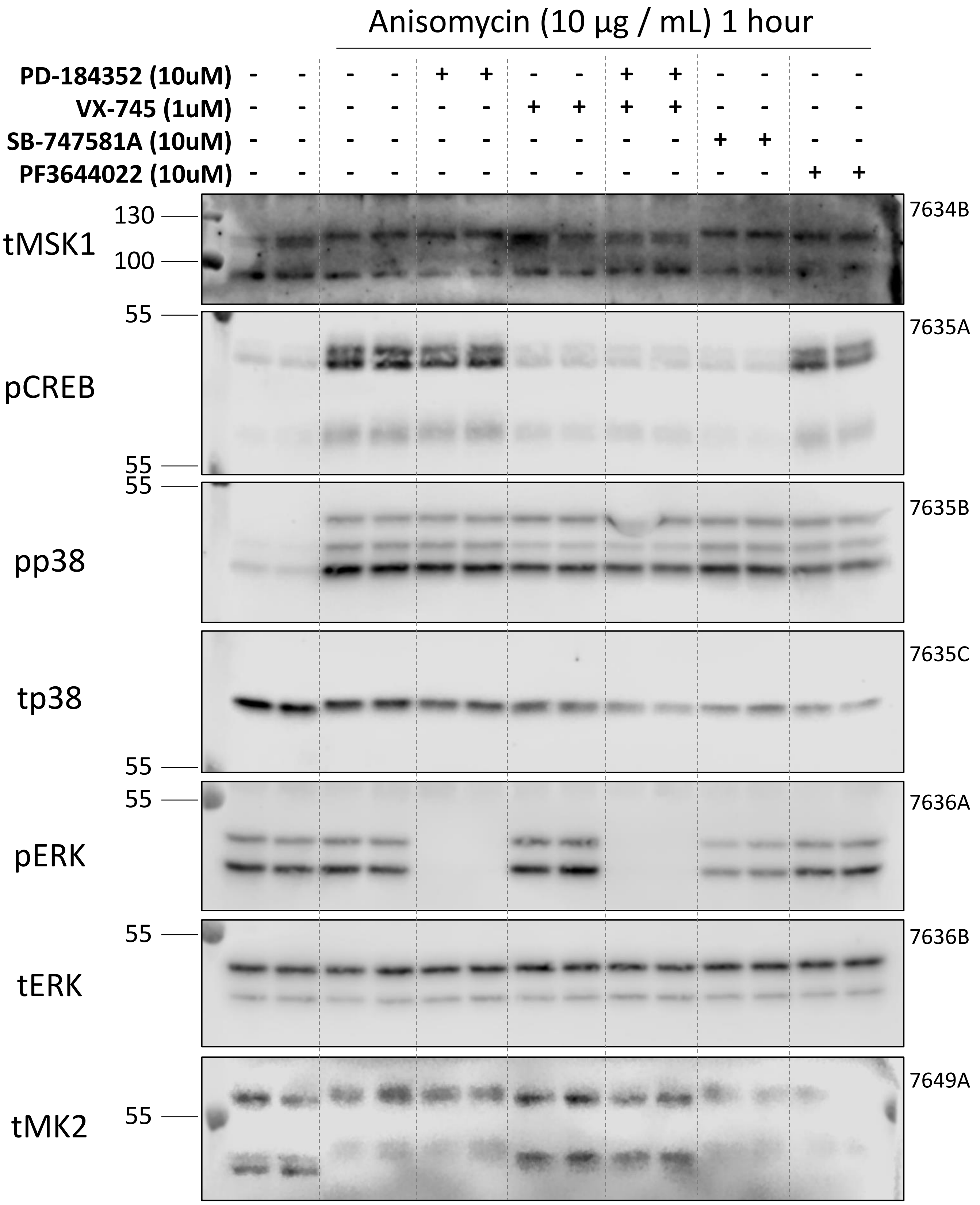

Supplementary Data S4

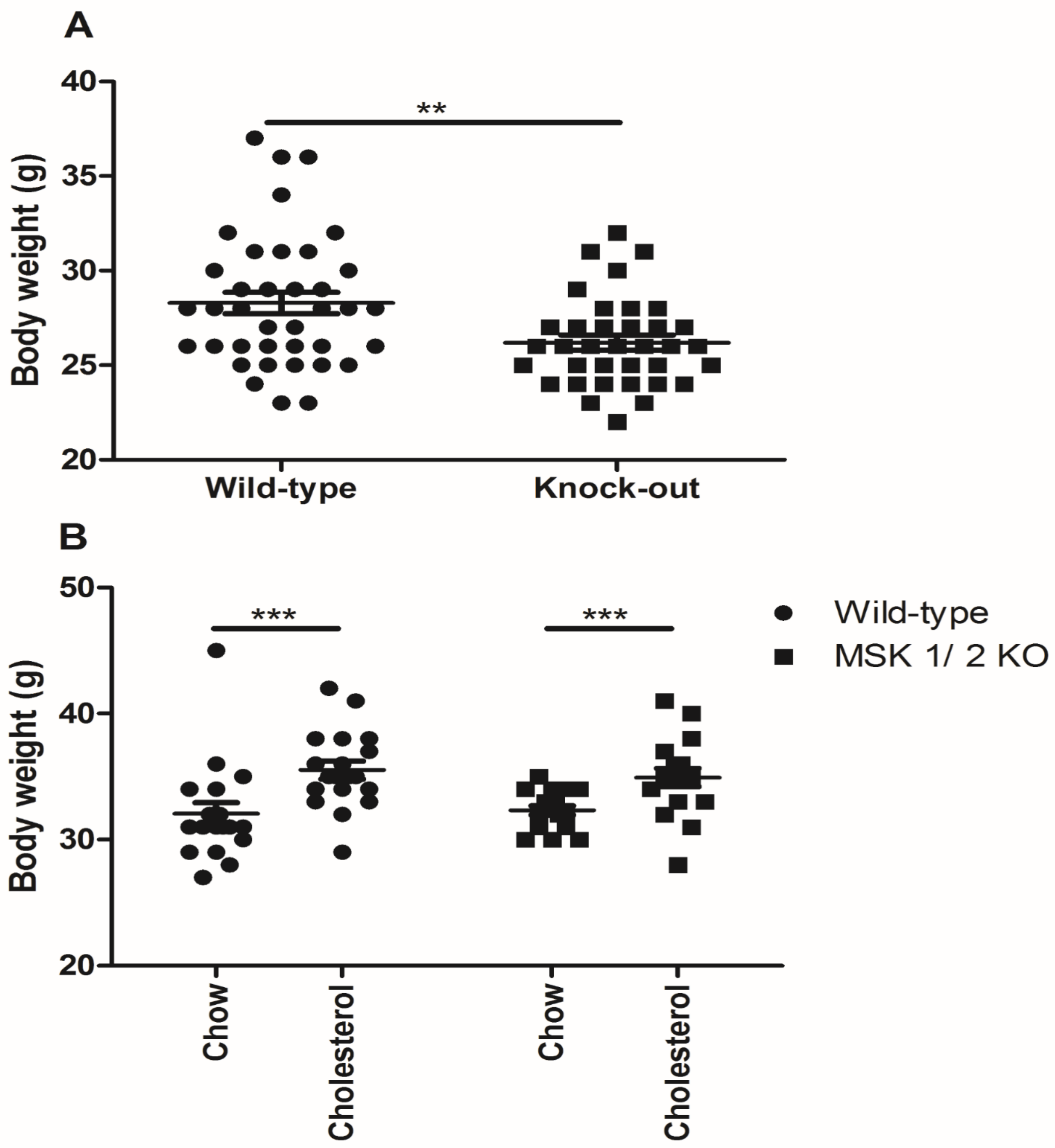

Supplementary Data S5

A

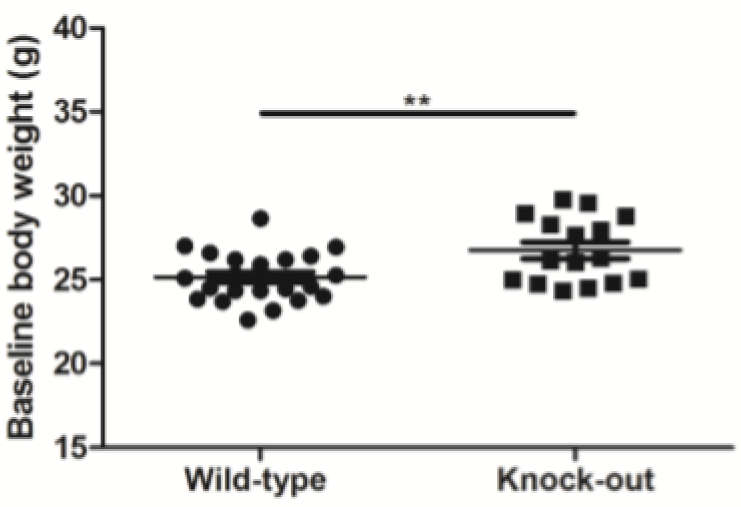

B

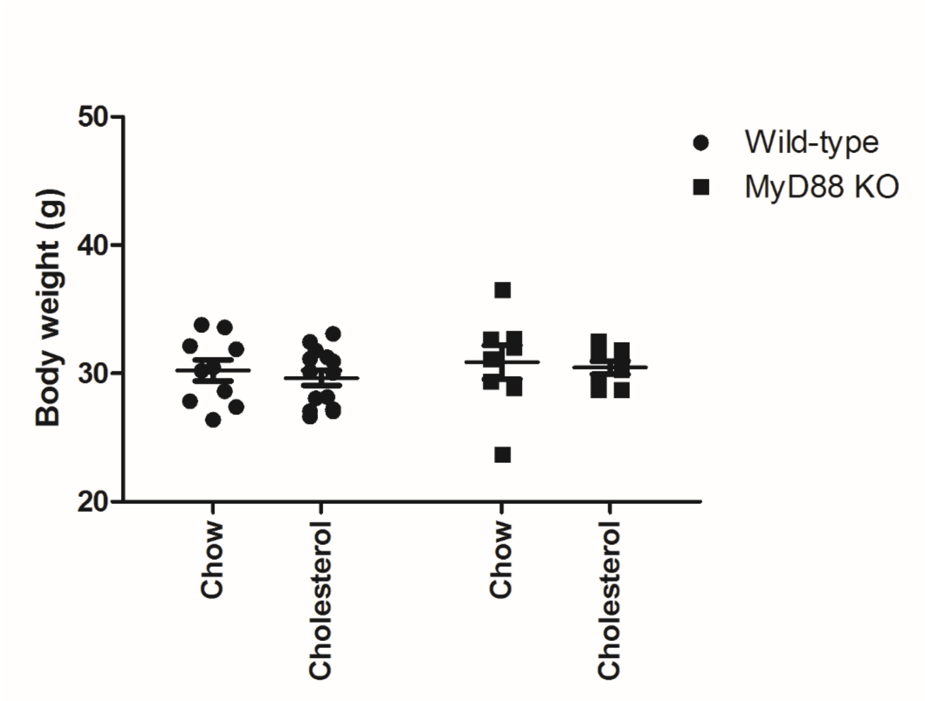

Table S1: STEMI patient and healthy volunteer characteristics

	**Healthy volunteers (N=28)**	**STEMI (N=35)**	**p value**
**Demographics**			
Age (Years)	60.0 (SD 10.5)	59.4 (SD 10.4)	>0.050[a]
Gender (% Male)	60.7	91.4	<0.010[b]
BMI (kg/m^2)	24.3 (SD 3.2)	28.1 (SD 5.5)	<0.010[a]
Systolic Blood Pressure (mmHg)	127.2 (SD 14.8)	117.0 (SD 14.8)	<0.010[a]
Diastolic Blood Pressure (mmHg)	77.0 (SD 8.9)	70.9 (SD 9.7)	<0.050[a]
Recruitment Delay (hours post PCI)	-	57.0 (SD 26.1)	-
**Past Medical History (%)**			
Diabetes Mellitus	-	16.7	-
Hypertension	-	37.5	-
Hypercholesterolemia	-	41.2	-
Angina/Myocardial Infarction	-	26.1	-
Stroke	-	-	-

**Smoking History (%)**			
Non-Smoker	78.6	17.4	<0.001[b]
Current	7.1	34.8	<0.010[b]
Ex-Smoker	14.3	47.8	<0.010[b]
**Diagnosis STEMI (%)**			
Anterior STEMI	-	25.7	-
Posterior STEMI	-	2.9	-
Inferior STEMI	-	42.9	-
Anteroposterior STEMI	-	2.9	-
Anterolateral STEMI	-	2.9	-
Infer posterior STEMI	-	8.6	-
Inferolateral STEMI	-	2.9	-
Posterior Inferolateral STEMI	-	5.7	-
Unclassified STEMI	-	5.7	-
**Echocardiography (%)**			
LV Systolic Dysfunction	-	70.6	-
Mild	-	54.2	-
Moderate	-	47.1	-
Severe	-	4.2	-

**Medications (%)**			
Aspirin	-	97.1	-
Ticagrelor/Clopidogrel	-	97.1	-
Statins	-	97.1	-
ACEi/ARBs	-	82.4	-
Beta Blockers	-	88.2	-
Anticoagulants	-	14.7	-
Calcium Channel Blockers	-	11.8	-
Diuretics	-	23.5	-

Table S2:

Taqman Primers for RT-qPCR analysis of peripheral blood mononuclear cells

Target	Probe / catalog Number	Supplier
RPS6KA5 (MSK1)	Hs01046591_m1	ThermoFisher Scientific
RPS6KA4 (MSK2).	Hs01071879_m1	ThermoFisher Scientific
ACTB	Hs01060665_g1	ThermoFisher Scientific
GAPDH	402869	ThermoFisher Scientific
18s	4333760T	ThermoFisher Scientific

Table S3:

Antibody details for western blot procedures

Target	Dilution	Supplier
phosphor-CREB at ser133	1/1000	Cell Signaling Technologies
phospho-p38 at Thr180/Tyr182	1/1000	Cell Signaling Technologies
p38-MAPK	1/1000	Cell Signaling Technologies
phospho-p44/42 MAPK (Erk1/2) at Thr202/Tyr204	1/1000	Cell Signaling Technologies
p44/42 MAPK (Erk1/2)	1/1000	Cell Signaling Technologies
phosphor-MAPKAPK-2 at Thr344	1/1000	Cell Signaling Technologies
MAPKAPK-2, phosphor-HSP27 at Ser 83	1/1000	Cell Signaling Technologies
MSK1	5 µg / mL	MRC PPU Reagents and Services (https://mrcppureagents.dundee.ac.uk/).
